# Intelligently learning from data

**DOI:** 10.1186/s13054-019-2424-7

**Published:** 2019-04-24

**Authors:** Edward Palmer, Roman Klapaukh, Steve Harris, Mervyn Singer, the INFORM-lab, Tim Bonnici, Ahmed Al-Hindawi, Tom Keen

**Affiliations:** 1 Bloomsbury Institute of Intensive Care Medicine, University College London, London, UK; 2 Research Software Development Group, Research IT Services, University College London, London, UK; 3 Critical Care, University College London Hospitals NHS Foundation Trust, London, UK

**Keywords:** Artificial intelligence, Machine learning, Statistical models

## Main text

Methods from the fields of artificial intelligence (AI) and machine learning (ML) are entering the medical literature at an unprecedented rate. A PubMed search using the keyword “Machine Learning” has shown an accelerating year-on-year increase in publications. Leveraged with “big data”, these approaches are often lauded as transformative in healthcare with the promise that they can and will solve all of our problems [1]. While these developments are indeed exciting, we caution the need to place realistic constraints on our expectations.

There is an established history of computational learning by fitting models to data. Previously the purview of statisticians, these models help us to understand complex problems by identifying patterns in data that are otherwise unnoticeable to humans. These models are typically associative in design, in that correlations do not necessarily imply causation. Given the well-described limitations of statistical models, there is a healthy scepticism of these approaches. Despite an awareness of these limitations, humans seem hard-wired to see a causal paradigm in mathematical models [2].

AI and ML models are a set of methods for learning patterns from data. Albeit optimised for different scenarios, statistical and ML approaches both share the same goal. AI models emphasise predictive accuracy, typically in large datasets, without a particular focus on inference for any one individual predictor. Statistical models stress a direct analytical approach, which characterises with uncertainty, estimators for individual predictors [3]. Statistical models tend to provide parameters that have a more directly interpretable human meaning. This ease of interpretability can make statistical models less able to describe complex phenomena. They are often either intractable at scale, or become powered to detect clinically meaningless signals. With these shortcomings in mind, AI models have enabled a new branch of learning from massive datasets.

AI models excel where they train on large volumes of high-quality labelled data. The prototypical example of which in medicine is computed tomography disease detection [4]. In these scenarios, predictive accuracy is the primary goal, and causal inferences are not necessary. Other examples of AI with direct relevance to this journal’s readership include early detection of the deteriorating patient [5] and strategies for fluid and inotropic administration in sepsis [6]. Not being conducted under a causal framework is a vital caveat to such approaches [7], yet our experience reveals a tendency for clinicians to draw causal conclusions from these models. While causal AI is a burgeoning field, generally AI models do not address the fundamental problem of causal inference, i.e. that one can only observe a single outcome for each patient (did the patient live or die), and not the counterfactual outcomes (how would the patient have responded under different treatments). Fundamentally, a model cannot learn from data that does not exist.

Judea Pearl, a professor of computer science and causal inference, describes AI models as running “almost entirely in an associational mode” [8]. Many of these approaches do not model the flow of time (that effect must follow cause) as associative models are true in either direction.

Spurious associations are commonplace when learning from data. These range from trivial (e.g. Facebook likes for “curly fries” are highly predictive of a high IQ [9]) to deeply concerning (e.g. asthma is predictive for a good outcome in pneumonia) [10]. AI models can be highly sensitive to the data on which they were trained, the addition of imperceptible noise, and improperly defined intermediate rewards (the means through which reinforcement models learn associations). This approach can lead to unexpected results, without a clear explanation of how or why this occurred [11, 12]. Komorowski et al. identify an optimal treatment strategy for sepsis that suggested less fluid administration than the human clinician. It is difficult to discern if this finding is causal (reducing fluid administration in septic shock will improve outcomes) or associational (better outcomes are seen for those patients who require less fluid). If these models are applied indiscriminately and without application of strong domain knowledge, spurious inferences are often found. Patients could potentially come to harm by extrapolating such findings to the bedside without a rigorous understanding of the causal pathways or underlying mechanisms that are typically discovered through experimental research.

Trusting and implementing AI models prematurely, just because they are “new” and therefore perceived as “better”, could lead to a lack of trust in these critical methods. The recent Topol review for the National Health Service [13] emphasised the importance of strengthening links between clinical practice and data science. As AI models enter the literature, and the medical community considers incorporating them into clinical decision-making tools, it is imperative that any outputs are both understood and challenged constructively. The rate at which new methods are appearing in the AI literature can leave little time to examine their limitations in a clinical context; impressive technical progress may outstrip the pace at which it is safe to implement.

Models of the real world, regardless of their origin, are still models. Models allow us to understand complex processes and, when safe and proper to do so, take actions based on their recommendations. AI models are compelling and provide rich insights into the world in which we practise. However, following either an AI model or statistical model without due care and consideration could place patients at risk.

## Abbreviations

AI: Artificial intelligence
IQ: Intelligence quotient
ML: Machine learning
